# Item

**Published:** 1909-09

**Authors:** 


### ITEM.

Messrs. Battle & Co., Saint Louis, recently issued No. 9 of their series of dislocation charts. These charts are sent free to physicians on request. The series includes illustrations in color of dislocations of the larger joints of the body.

